# Plasma Cell Leukemia: A Review of 3 Cases Managed in Kenya

**DOI:** 10.1155/2021/4843818

**Published:** 2021-07-27

**Authors:** Matilda Ong'ondi, Elizabeth Kagotho

**Affiliations:** ^1^Department of Internal Medicine; Hemato-oncology Unit, Kenyatta National Hospital, Nairobi, Kenya; ^2^Department of Pathology and Laboratory Medicine, Aga Khan University hospital, Nairobi, Kenya

## Abstract

Plasma Cell Leukemia (PCL) is a rare and aggressive form of plasma cell dyscrasia that can arise either de novo (primary plasma cell leukemia) or evolve from previously diagnosed and treated multiple myeloma (secondary PCL). We highlight three clinical cases with very different presentations as a reminder of this diagnosis. The cases also highlight the diversity and variability that cover a patient's journey that is highly dependent on accessibility based on financial capability and social support. The clinical presentation is more aggressive due to the higher tumour burden and more proliferative tumor cells with cytopenias being profound and more organomegaly. The diagnosis is made based on at least 20% of total white blood cells being circulating plasma cells with a peripheral blood absolute plasma cell count of at least 2 × 10^9^/l. Treatment with novel agents followed by autologous stem cell transplant in those who are transplant eligible leads to better outcomes.

## 1. Introduction

Plasma Cell Leukemia (PCL) is a rare and aggressive form of plasma cell dyscrasia that can arise either de novo (primary plasma cell leukemia) or evolve from previously diagnosed and treated multiple myeloma (secondary PCL) [1]. These are seen as distinct entities that vary in clinical presentation and survival. Due to the rarity of this clinical entity, most of the data are case reviews and retrospective studies. We report 3 clinical cases recently managed and discuss briefly the available data around this condition.

## 2. Case Discussions

### 2.1. Case 1

A 51-year-old female with no comorbidities was diagnosed with primary plasma cell leukemia in July 2018 after presenting with symptomatic anemia. She also reported headaches with no focal neurological deficits and had right lower limb swelling not associated with trauma or skin lesions. On physical examination, the only remarkable finding was pallor and right calf swelling with no signs of cellulitis. A complete blood count revealed a leucocytosis of 99,000/ul, normocytic anemia of 7.9 g/dl, and thrombocytopenia of 68,000/ul. A peripheral blood film revealed 79% plasma cells, and bone marrow aspirate showed plasmacytosis of 89% with serum protein electrophoresis having an M protein of 6 g/l. The serum creatinine was slightly elevated at 158 umol/l, and calcium levels were normal with an elevation of lactate dehydrogenase at 469 U/l. A Doppler ultrasound of the right lower limb ruled out deep venous thrombosis but found a fluid collection suspected to be a hematoma. A few days into her first admission at the referral hospital, she complained of worsening headaches with associated blurring of vision and vomiting. Computed Tomography (CT) of the brain performed showed bilateral frontoparietal and temporal region subacute subdural hematomas. She was managed conservatively for the hematomas and prophylactic low-molecular-weight heparin (enoxaparin) held.

**Figure 1:**
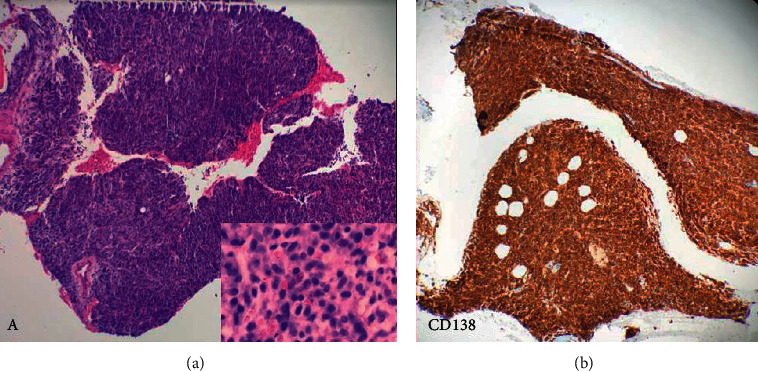
Representative micrograph of the bone marrow biopsy (a, b); original magnification ×20 haematoxylin eosin (H&E) stain shows a diffuse infiltration by plasma cells with suppressed trilineage haematopoiesis. These plasma cells are positive for CD138 and negative for CD56.

Initial definitive treatment was commenced with cyclophosphamide, thalidomide, and dexamethasone based on the available medications; she received 5 cycles. On reassessment, there was clearance of peripheral blood plasmacytosis on the peripheral smear; however, bone marrow biopsy had persistent bone marrow plasmacytosis of 90% with immunohistochemistry stains being positive for CD138, CD 20, MUM 1, and BCL 2 with negative CD56, as shown in Figure 1.

Her treatment regimen was changed to bortezomib, lenalidomide, and dexamethasone with complete response after 4 cycles, no M protein, and normal serum free light chains with bone marrow biopsy with immunohistochemistry showing a plasmacytosis of less than 5%. She proceeded to go for autologous stem cell transplant out of country in May 2019.

Evaluation at the transplant facility included a bone marrow aspirate and trephine that revealed a plasmacytosis of 2% with no peripheral blood plasma cells. Other blood parameters such as total blood count and liver and kidney tests were normal, and a PET CT showed extensive lytic lesions. She received 2 more doses of bortezomib and dexamethasone as she awaited pretransplant work up.

She was mobilized with plerixafor after unsuccessful attempts with Granulocyte Colony-Stimulating Factor (GCSF), and conditioning was with melphalan at 200 mg/m2 followed by infusion of stem cells 24 hours later. Her posttransplant period was complicated by mucositis and febrile neutropenia that responded well to broad-spectrum antibiotics. She had minimal signs of engraftment syndrome managed with steroids and achieved engraftment of white cells and platelets by day 11. After normalization of counts and full recovery, she was discharged back home to have consolidation chemotherapy after 60days after transplant (twice weekly bortezomib and dexamethasone with 21 days of lenalidomide 10 mg) and then proceed to maintenance with lenalidomide and dexamethasone. The patient did well and remained in remission for 1 year.

### 2.2. Case 2

**Figure 2:**
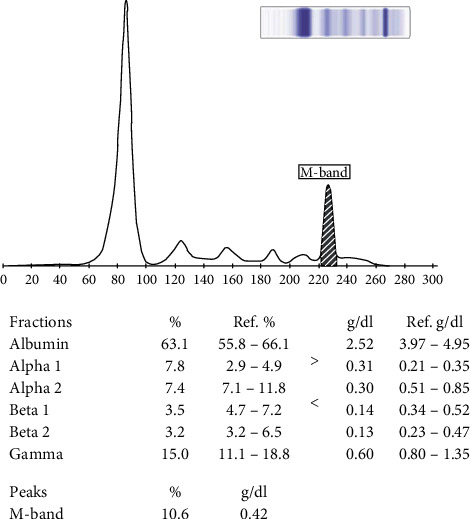
Serum protein electrophoresis showing an M protein of 4.2 g/l.

A 48-year-old male patient presented with generalized body weakness and unintentional weight loss with no fever or bleeding diathesis. Upon admission, he was found to have pallor but no palpable peripheral adenopathy or hepatosplenomegaly. A total blood count revealed a macrocytic anemia (Hb 7.9 g/dl, MCV 106 fl) with a leucocytosis of 36,290/ul (neutrophils 26%, lymphocytes 20%, and monocytes 51%) with a platelet count of 141,000/ul. Reticulocyte count was low at 1% with normal serum B12 and folate levels. A peripheral smear revealed anisocytosis, normochromic red cells, left shift of white blood cells, and large mononuclear plasmacytoid cells which were more than 20%. A diagnosis of plasma cell leukemia was made after bone marrow aspiration that showed infiltration with plasma cells and increase in small mature lymphocytes; however, there was no trephine with immunohistochemistry. In addition, flow cytometry on peripheral blood showed that the abnormal cell population expressed CD38, CD138 and they were lambda restricted; however, B- and T-cell markers were negative as well as CD56. Serum protein electrophoresis showed an M band of 4.2 g/l, as shown in Figure 2, with elevated lambda free light chains of 461 mg/l. Serum immunofixation was not performed in this case. CT scans of the chest and abdomen were unremarkable with no adenopathy and normal-size spleen and liver. Serology for HIV, hepatitis B, and hepatitis C was negative.

He was transfused during his first admission, discharged home, but was readmitted one week later with worsening generalized body weakness, dyspnea, and diarrhoea. He was sick looking and markedly dehydrated with bilateral pitting edema to the level of the knee but clear chest exam. He was hypotensive with a blood pressure of 84/37 mmHg with hypoxia of 87% room air but afebrile at 36.7°C, and a diagnosis of severe sepsis due to gastroenteritis was made and he transferred from the emergency unit to the high-dependency unit. His haemoglobin level was 11 g/dl given recent transfusion but worsening of leucocytosis (60,000/ul) and thrombocytopenia of 43,000/ul. He had acute kidney injury with low total protein and serum albumin (Table 1 outlines a summary of a few selected results during his hospital stay).

The second admission was more protracted lasting 30 days with a predominant time being spent in the high-dependency unit (HDU). Due to lack of blood pressure response to fluid resuscitation, he was initiated on inotropic support. Hypoalbuminemia resulted in third spacing of fluid, but this improved with administration of intravenous albumin. Given the aggressiveness of disease with declining haemoglobin and platelet count, he received adjusted-dose chemotherapy: 1 gram of intravenous cyclophosphamide and weekly dexamethasone 40 mg with bortezomib being held due to low platelets. He tolerated his chemotherapy well. However, on hospital day 10, he developed worsening respiratory symptoms with a new oxygen requirement, and chest radiograph revealed features of pneumonia for which he was commenced on broad-spectrum antibiotics with piperacillin-tazobactam. Subsequently, on hospital day 15, he further desaturated with clinical signs of a right-sided pleural effusion. This was hemorrhagic and drained with a pigtail catheter with the fluid analysis showing an exudate, normal adenine deaminase levels, negative Zielh Niessen stain, and no malignant cells on cytology. This was, therefore, managed as a parapneumonic effusion and drain removed after having no more output. He required transfusions whenever his haemoglobin dropped but did not need platelet transfusion.

He slowly improved initially getting off inotropes, weaning off oxygen successfully to the point of discharge after 30 days in the hospital.

Two weeks after discharge, the patient was more stable with good rehabilitation given prolonged hospitalization that was associated with significant weight loss and reduced level of activity. He was commenced on a bortezomib-based regimen (bortezomib, lenalidomide, and dexamethasone) and has achieved complete response with a plan for autologous stem cell transplant to consolidate the response.

### 2.3. Case 3

A 53-year-old female referred with a 9-month history of easy fatigability. She had associated bone pains, predominantly back and knee pain, which were previously managed as arthritis. She had no bleeding diathesis, hematemesis, or melena. There was no known family history of malignancy or haematological condition, and she had no history of tobacco use or exposure to chemicals and reported no underlying comorbidities.

She presented to a health facility with palpitations and worsening general body weakness. There, she was found to have severe anemia (haemoglobin of 4.5 g/dl), thrombocytopenia, and acute kidney injury with high serum calcium levels. Her record of actual values was not available. She was transfused both packed red blood cells and pooled platelets. In addition, she received intravenous fluids which resulted in improvement of her renal function. Other laboratory investigations revealed normal serum folate and B12 levels, elevated uric acid, and lactate dehydrogenase (LDH) and a total protein of 100 gm/dl with a monoclonal protein of 54 g/l. An abdominal ultrasound revealed splenomegaly but no intra-abdominal adenopathy. A peripheral blood film showed a leucoerythroblastic picture with atypical mononuclear cells with plasmacytoid appearance at 45% and bone marrow aspirate and trephine revealed infiltration with 74% atypical plasmacytoid cells with a limited immunohistochemistry stains being positive for CD38 and negative for CD20, CD3, MUM1/IRF4, and Tdt.

Her initial treatment course comprised steroids and allopurinol while receiving transfusions of blood products and later was started on bortezomib, lenalidomide, and dexamethasone. Bortezomib was held after a few treatments due to low platelet counts and challenges of platelet transfusions; she, therefore, continued with low-dose lenalidomide and dexamethasone. Two months into her treatment, she developed congestive heart failure with preserved ejection fraction on echocardiography and symptoms improved on diuretics. She had treatment interruptions due to congestive hepatopathy resulting in transaminitis, and medications were restarted when this improved. She also continued to fluctuate in her transfusion requirements which would be carried out at remote facilities due to financial constraints. Initially, she did well; however, during her third cycle, she was reported to have easy fatiguability and shortness of breath and taken to a nearby hospital for urgent care; however, she succumbed in a few days. The cause of death at this point was not clear given lack of access to records from the admitting hospital.

This case highlights the challenges that clinicians in sub-Saharan Africa face in managing patients where cost affects choices patients make in seeking care, especially with regard to hospital admissions. As a result, in the clinical journey, they receive part of care at a facility away from their primary doctor, especially in-patient.

## 3. Epidemiology and Clinical Presentation

PCL is not common with various studies reporting incidence of less than 1% to 4% [2–4]. The median age is younger than for MM patients, between 50 and 60 years with a slight male predominance [5, 6]. Studies conducted in China revealed a younger population below 50 years [7, 8]. Our patients were all less than 60 years with the youngest being 48 years.

Unlike MM, patients with plasma cell leukemia have a more aggressive clinical presentation due to a higher tumour burden. The symptoms may range from profound cytopenias, mainly anemia and thrombocytopenia resulting in bleeding diathesis. They can also present with bone pain, symptoms due to hypercalcemia, or acute kidney injury [5, 9, 10].

On physical examination, these patients have a higher prevalence of organomegaly with involvement of the liver and spleen [11]. Other patients may have pleural effusion, neurological deficits due to CNS involvement, or palpable extramedullary soft tissue plasmacytomas. This is reflected by cases discussed.

Lytic lesions on imaging is reported to be lower than in MM; however, it may be found in approximately half of the patients more so those with sPCL [11]. This is illustrated in the first case where the patient had extensive lytic disease, hence the question whether she had sPCL as opposed to pPCL.

## 4. Diagnosis

Kyles' criteria described in 1974 required at least 20% of total white blood cells be circulating plasma cells with a peripheral blood absolute plasma cell count of at least 2 × 10^9^/l [12]. There is debate on whether both criteria need to be met as per Kyle et al.'s recommendation, and many reports have used the presence of at least one criteria, although there is no consensus as to threshold for diagnosis, with the increasing studies showing that patients with circulating plasma cells between 5 and 20% had shorter median overall survival of approximately 6 months, similar to those with >20% PCs.

Advances in flow cytometry have allowed for better characterization and clonal assessment of plasma cell population. Clonality is important to establish to rule out reactive plasma cells in context of infection or inflammation which are polyclonal or exclude other lymphoproliferative diseases such as lymphoplasmacytic lymphoma [9]. The International Myeloma Working Group (IMWG) and the World Health Organization (WHO) have accepted that either one of the criteria is sufficient.

CD138 and CD38 are expressed in plasma cells in multiple myeloma and plasma cell leukemia. CD56, a neuronal cell adhesion molecule, seems to be more prevalent in MM as opposed to B-cell marker CD 20 which are more positive in PCL. CD56 which facilitates interactions with the bone marrow microenvironment and prevents circulation from the bone marrow to the blood tends to be absent in PCL [13]. In addition, there is lower expression of CD9, CD117, and HLA DR. There is higher CD28 expression in secondary PCL which correlates with a rise in plasma cell proliferation and disease progression. Increased expression of CD27 has been associated with activation of the antiapoptotic pathway. The IMWG consensus on PCL is that measurement of immunophenotypic residual disease is needed when there is no evidence of plasma cell infiltration on routine morphology evaluation.

A majority of patients will have markedly elevated serum lactate dehydrogenase (LDH) levels and B_2_ macroglobulin [1, 14].

Most patients with PCL tend to have hypodiploidy or pseudodiploidy and high risk or complex cytogenetic abnormalities [5, 8, 15]. Majority of the PCL cases had translocation involving the immunoglobulin heavy-chain locus (IgH) on 14q32 such as t (11, 14), t (4; 14), and t (14; 16). TP53 inactivation, MYC translocations, and mutations in K-RAS and N-RAS were among those documented with PTEN deletion which cause Akt activation being more in sPCL.

Imaging either by whole-body MRI or PET CT scan is important to check for any extramedullary component [1].

## 5. Management

Management includes adequate supportive care as well as definitive therapy that depends on the transplant eligibility. Choice of regimen will depend on patient performance status, existing comorbidities, and availability of drugs, as well as the centres' ability to administer the regimen.

Supportive treatment includes, but is not limited to, transfusions, treating hypercalcemia, renal insufficiency, and infections. It is important to watch out for tumour lysis given the high tumour burden in this patient population. This would require the managing team to monitor uric acid, potassium, calcium, phosphorous, and creatinine levels at the time of instituting definitive therapy.

Studies have shown that use of drug combinations involving novel agents has better responses and median overall survival than use of single agents or conventional chemotherapy [16]. The use of novel agents (immunomodulators and proteasome inhibitors) has been associated with a higher response rate which is consolidated to an even deeper response by autologous stem cell transplant resulting in improved overall survival [17]. Some examples of regimes in transplant candidates are VTD-PACE (bortezomib, thalidomide, dexamethasone, cisplatin, doxorubicin, cyclophosphamide, and etoposide), HyperCVAD-VTD (hyperfractionated cyclophosphamide, vincristine, adriamycin, dexamethasone, bortezomib, thalidomide, and dexamethasone), PAD (Bortezomib, Adriamycin, and Dexamethasone), and VCD (bortezomib, cyclophosphamide, and dexamethasone). After transplant, it is highly recommended that these patients receive consolidation therapy with bortezomib-based combinations followed by maintenance.

Nontransplant candidates also receive bortezomib-based regimens so as to achieve a rapid response and improve outcome. These include CyBorD (cyclophosphamide, bortezomib, and dexamethasone), VTD (bortezomib, thalidomide, and dexamethasone), and MPV (melphalan, bortezomib, and prednisone) among others [18].

Many studies have demonstrated the effectiveness of bortezomib in clearing plasma cell leukemia, normalizing platelet count, and better overall survival compared to conventional therapies [13, 19, 20]. Musto et al. showed 92% overall response with the use of bortezomib which was also replicated by the Greek Myeloma group that looked at a larger patient population [16, 19]. It is, therefore, important that where patients have no contraindications, bortezomib-based regimens be used.

Lenalidomide-based therapies have reported overall responses of 60% with clear lack of benefit with thalidomide. The later should, therefore, be avoided as initial therapy and especially as monotherapy [19, 21–23].

An Italian multicentre retrospective review of 128 PCL patients showed that hematopoietic stem cell transplant improves overall survival and duration of response by 69% and 88%, respectively [6]. Results from the Center for International Blood and Marrow Transplant Research showed that autologous stem cell transplant is better than allogeneic stem cell transplant due to higher nonrelapse mortality with no benefit in overall survival [21]. The current challenge is the short duration of response as demonstrated by a large cohort of 272 pPCL patients in the European Group for Blood and Marrow Transplantation when compared to myeloma [24]. Newer agents have also been used in plasma cell leukemia such as carfilzomib, ixazomib, monoclonal antibodies to CD38 such as daratumumab, and novel therapies such as Bcl-2 inhibitor, venetoclax in those with t(11; 14) [25, 26].

## 6. Prognosis

Plasma cell leukemia has a poor prognosis in comparison to multiple myeloma [11, 14, 26]. In addition, the outcome for sPCL is much worse and most studies report survival of few months in comparison to pPCL [9, 12, 14, 27]. Talamo et al. who studied the outcome of PCL in the era of novel agents found a median survival of 21 months for pPCL in comparison to 4 months for sPCL which was statistically significant (*p*=0.015) [27].

The Center for International Blood and Marrow Transplant Research documented an overall survival of 51% at 5 years after pPCL treatment had been consolidated with autologous stem cell transplant [21].

Studies have demonstrated some factors such as age more than 60 yrs, platelet count less than 100,000/ul, and peripheral blood plasma cells more than 20% to be predictors of worse survival [17]. Others reported include poor performance status (ECOG >2) and elevated serum LDH, as well as B2 microglobulin and cytogenetics such as 17 p deletion [7].

## 7. Conclusions

Plasma cell leukemia is a rare aggressive form of plasma cell dysrasia that needs to be diagnosed early and managed aggressively before patients get complications. Given poor prognosis, it is important to use novel agents as part of the induction regimen followed by autologous transplant in those who are transplant eligible. Despite the improvement in diagnostics and treatment, sub-Saharan Africa faces challenges where the patients' treatment journey depends on what they are able to afford and accessibility to those services.

## Figures and Tables

**Table 1:** Selected lab investigations during his hospital stay.

Investigations	Baseline	Day 4	Day 9	Day 13	Day 15	Day 16
Hemoglobin (g/dl)	11.3	9.9	9.3	11.6	12.9	12.3
White cell count	60.2	44.98	25.35	12.13	13.48	17.34
Platelet	43	36	31	76	86	161
Urea (1.5–8.5 mmol/l)	8.95	6.97	5.41	3.88	4.09	3.6
Creatinine (40–133 umol/l)	154	148	80	58	53	60
K (3.5–5.5 mmol/l)	4.15	4.25	3.48	3.23	3.8	4.18
T protein (60–83 gm/l)	39.8	41.9	46.8	57.3	53.8	—
Albumin (35–52 g/l)	24.27	27	27.6	43.56	37.8	—
ALT (0–41 u/l)	30.6	40.9	53.6	31.5	21	—
Total bilirubin(2–21 umol/l)	37	33	45	45	23	—
LDH (90–244 u/l)	1116	—	—	738	—	—
Uric acid (202–416 umol/l)	399	122	119	220		
Calcium (2.02–2.6 mmol/l)	2.2	—	2.2	—	—	—
